# Synthesis and characterization of polysaccharide-maghemite composite nanoparticles and their antibacterial properties

**DOI:** 10.1186/1556-276X-7-576

**Published:** 2012-10-22

**Authors:** Simona Liliana Iconaru, Alina Mihaela Prodan, Mikael Motelica-Heino, Stanislas Sizaret, Daniela Predoi

**Affiliations:** 1 National Institute of Materials Physics, 105 bis Atomistilor, P.O. Box MG 07, Bucharest-Magurele, 077125, Romania; 2 Carol Davila University of Medicine and Pharmacy, 8 Eroii Sanitari, sector 5, Bucharest, Romania; 3 Emergency Hospital Floreasca, Bucharest 5, Calea Floresca nr 8, sector 1, Bucharest, Romania; 4 ISTO, Université d’Orléans, Orléans cedex 02, 45067, France

**Keywords:** Iron oxides, Biological polymers, Antibacterial activity

## Abstract

The aim of this study was to obtain saccharide (dextran and sucrose)-coated maghemite nanoparticles with antibacterial activity. The polysaccharide-coated maghemite nanoparticles were synthesized by an adapted coprecipitation method. X-ray diffraction (XRD) studies demonstrate that the obtained polysaccharide-coated maghemite nanoparticles can be indexed into the spinel cubic lattice with a lattice parameter of 8.35 Å. The refinement of XRD spectra indicated that no other phases except the maghemite are detectable. The characterization of the polysaccharide-coated maghemite nanoparticles by various techniques is described. The antibacterial activity of these polysaccharide-coated maghemite nanoparticles (NPs) was tested against *Pseudomonas aeruginosa* 1397, *Enterococcus faecalis* ATCC 29212, *Candida krusei* 963, and *Escherichia coli* ATCC 25922 and was found to be dependent on the polysaccharide type. The antibacterial activity of dextran-coated maghemite was significantly higher than that of sucrose-coated maghemite. The antibacterial studies showed the potential of dextran-coated iron oxide NPs to be used in a wide range of medical infections.

## Background

The progress in the field of nanoscale science and engineering provides us with unprecedented understanding and control of matter at atomic and molecular levels. The recent advances in nanotechnology allow us to fabricate more and more advanced materials with unusual magnetic, electric, optical, and biological properties [1].

Nanotechnology develops new research on nanostructured materials [2] in order to answer the yet unsolved problems in various areas such as environment, medicine, biology, chemistry, and electronics [3-5]. In the field of biomedical application, outstanding progresses involving magnetic nanoparticles have been made due to their unique properties at nanometric scale [6,7]. Nanoparticles have physicochemical properties that are characteristic of neither the atom nor the bulk counterpart [8]. The size of these nanoparticles makes them ideal for nanoengineered surfaces and for the production of functional nanostructures. Those abilities make them suitable for use in biomedical applications such as contrast agents in MRI [9], targeted drug delivery in tumor therapy, hyperthermia, catalysis, biological separation, biosensors, and diagnostic medical devices [10,11].

The iron oxide magnetite (Fe_3_O_4_) and its oxidized form maghemite (γ-Fe_2_O_3_) are the most studied magnetic particles in medicine and biotechnology because of their unique magnetic properties and biocompatibility at nanometric scale [12,13]. The important properties of magnetic particles for biological and biomedical applications are controllable shape (for biological and biomedical purposes, they must have spherical shape), biocompatibility, nontoxicity, narrow and moderate size distribution, high crystallinity, large surface areas (for maximal protein or enzyme binding), and the ability to well disperse in an aqueous medium [14-17]. In order to meet these high demands, in the recent years, intense studies involving the means to develop a stable, nontoxic, and cheap way to obtain magnetic nanoparticles with the abovementioned parameters [18] have been made. Besides those demands, nowadays, studies revealed the need for these particles to exhibit antibacterial properties in order to be successfully used in environmental and biomedical applications [19].

Nowadays, antibiotic resistance has become a serious public health problem. As a result of the resistance to the existing portfolio of antimicrobial drugs, there is an increasing need to design new antibacterial and antifungal agents with better activity profiles and lower toxicity [20]. Nanotechnology is expected to open some new ways to fight and prevent diseases using atomic scale-tailored materials [21]. Bio-nanotechnology investigates the interactions between nanoscale materials and biological systems and creates the technologies for interfacing the two. The insertion of prosthetic medical devices for different exploratory or therapeutical purposes, especially in severe pathological conditions, represents a risk factor for the occurrence of chronic infections in developed countries, being characterized by slow onset, middle-intensity symptoms, chronic evolution, and resistance to antibiotic treatment [22]. The microbial species of clinical interest, often involved in biofilm-associated diseases, belong to a very large spectrum, from the Gram-positive (*Staphylococcus epidermidis* and *Staphylococcus aureus*) to the Gram-negative (*Pseudomonas aeruginosa* and *Escherichia coli*) pathogens and to the different members of the genus *Candida *[23]. Iron oxide-based magnetic nanoparticles have been widely used in a variety of biomedical applications such as magnetic separation, magnetic resonance imaging, hyperthermia, magnetically guided drug delivery, tissue repair, and molecular diagnostics [24].

The aim of this article is to integrate and extend information from journal literature about antibacterial properties of iron oxide coated with different saccharides. In this work, polysaccharide-coated iron oxide nanoparticles were synthesized by an adapted coprecipitation method. The obtained polysaccharide-coated maghemite nanoparticles were systematically investigated by X-ray diffraction and transmission electron microscopy (TEM). The magnetic hysteresis cycles for the powder samples were also determined at room temperature. The antibacterial activity of these polysaccharide-coated maghemite nanoparticles (PMC-NPs) was tested against *P. aeruginosa* 1397, *Enterococcus faecalis* ATCC 29212, *Candida krusei* 963, and *E. coli* ATCC 25922.

## Methods

### Materials

Dextran (H(C_6_H_10_O_5_)_x_OH; MW ~ 40,000) and sucrose (C_12_H_22_O_11_) were purchased from Merck (Whitehouse Station, NJ, USA). Iron dichloride tetrahydrate (FeCl_2_·4H_2_O), iron trichloride hexahydrate (FeCl_3_·6H_2_O), sodium hydroxide (NaOH), potassium dichromate (K_2_Cr_2_O_7_), hydrochloric acid (HCl), and perchloric acid (HClO_4_) were also purchased from Merck. Deionized water was used in the synthesis of nanoparticles.

### Synthesis of dextran-coated iron oxide

Iron dichloride tetrahydrate (FeCl_2_·4H_2_O) in 2 M HCl and iron trichloride hexahydrate (FeCl_3_·6H_2_O) were mixed at 90°C (Fe^2+^/Fe^3+^ = ½). The mixture was dropped into dextran (20 g in 100 mL of water) and 200 mL of NaOH (2 mol·L^−1^) solution under vigorous stirring for about 30 min. The resulting solution was heated at 90°C for 1 h with continuous agitation (200 rotations/min). The 5 M NaOH was added dropwise to obtain a pH of 11. The precipitate was centrifuged and treated repeatedly with perchloric acid (3 mol·L^−1^) solution until the Fe^2+^/Fe^3+^ ratio in the solid was approximately 0.05. After the last separation by centrifugation, the particles were dispersed into dextran (20 g in 100 mL of water). The product was dried at 40°C (dextran-coated iron oxide (DIO)-NP samples).

### Synthesis of sucrose-coated iron oxide

The sucrose solution (20 g in 100 mL of water) was heated at 90°C for 1 h with continuous agitation (200 rotations/min), and 200 mL of NaOH (2 mol·L^−1^) solution was added slowly. FeCl_2_ and FeCl_3_ (Fe^2+^/Fe^3+^ = ½) mixed at 90°C were dropped into the solution. The 5 M NaOH was added dropwise to obtain a pH of 11. The suspensions were then heated at 90°C for 1h with continuous agitation (200 rotations/min). The precipitate was centrifuged and treated repeatedly with HClO_4_ (3 mol·L^−1^) solution until the Fe^2+^/Fe^3+^ ratio in the solid was approximately 0.05. After the last separation by centrifugation, the particles were dispersed into dextran (20 g in 100 mL of water). The product was separated by centrifugation (10,000 rpm) and dried at 40°C (sucrose-coated iron oxide (SIO)-NP samples).

In the current experiment, K_2_Cr_2_O_7_ is used as the titrant to determine the amount of iron. The contents of Fe^2+^ and Fe^3+^ are determined by a common titration method after dissolution in concentrated hydrochloric acid. Fe^2+^ was titrated potentiometrically with K_2_Cr_2_O_7_. The total Fe content was determined by the same way after reduction of iron by SnCl_2_.

### Characterization

X-ray diffraction measurements were recorded using a Bruker D8 Advance diffractometer (Madison, WI, USA), with Cu K_α_ radiation, and a high-efficiency one-dimensional detector (LynxEye type) operated in integration mode. The diffraction patterns were collected in the 2*θ* range of 20° to 70°, with a step of 0.02° and a 34-s measuring time per step. TEM studies were carried out using a JEOL 200 CX (Akishima-shi, Japan). The specimen for TEM imaging was prepared from the particle suspension in deionized water. A drop of well-dispersed supernatant was placed on a carbon-coated 200-mesh copper grid, followed by drying the sample at ambient conditions before it was attached to the sample holder on the microscope. The magnetic properties of the samples were measured using a superconducting quantum interference device (MPMS magnetometer, Quantum Design, San Diego, CA, USA) at room temperature.

### The *in vitro* antibacterial activity

#### Assessment of the antimicrobial and anti-pathogenic activity of the new oxides

The *in vitro* qualitative screening of the antimicrobial activity was carried out by an adapted agar diffusion technique using a bacterial suspension of 0.5 McFarland density obtained from 24-h cultures. The antimicrobial activities of the newly synthesized compounds were determined against clinical and American Type Culture Collection (ATCC) reference microbial strains, i.e., *P. aeruginosa* 1397, *E. faecalis* ATCC 29212, *C. krusei* 963, and *E. coli* ATCC 25922.

The microbial strain identification was confirmed using the VITEK 2 automatic system (bioMérieux, Inc., Durham, NC, USA). VITEK cards for identification and susceptibility testing (GNS-522) were inoculated and incubated according to the manufacturer's recommendations. The results were interpreted using the software version AMS R09.1. The compounds were solubilized in dimethyl sulfoxide to a final concentration of 10 mg/mL. A volume of 10 μL of each tested compound solution was distributed directly on the solid medium previously seeded with the microbial inocula. The inoculated plates were incubated for 24 h at 37°C. Antimicrobial activity was assessed by measuring the growth inhibition zone diameters expressed in millimeters [25-27]. Following the results of the qualitative screening, only the microbial strains which proved to be susceptible have been further tested in the quantitative assay.

The quantitative assay of the minimal inhibitory concentration (MIC; μg/mL) was based on liquid medium twofold microdilutions performed in 96-multi-well plates. For this purpose, serial binary dilutions of the tested compounds (ranging between 0.01 and 5 μg/mL) were performed in a 200-μL volume of nutrient broth/YPG, and each well was seeded with 20 μL of microbial inocula of 0.5 McFarland density. The plates were incubated for 24 h at 37°C for bacterial strains and for 48 h at 28°C for fungal strains, and MICs were macroscopically read as the last concentration of the compound which inhibited microbial growth and by measuring the absorbance of the obtained culture at 620 nm [25-27].

## Results and discussion

**Figure 1:**
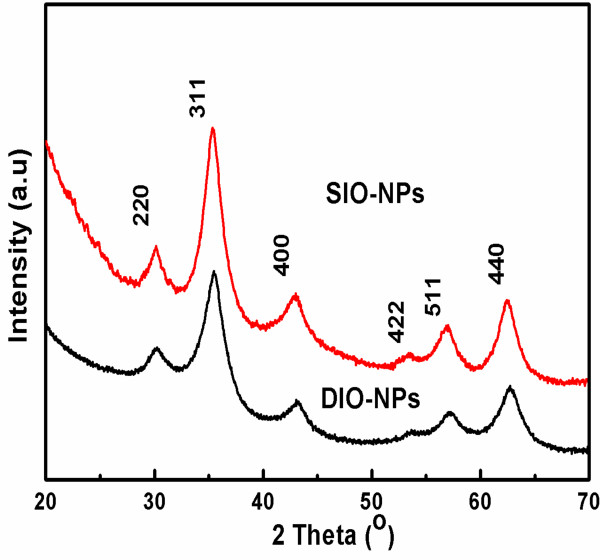
X-ray diffraction pattern of synthesized DIO-NPs and SIO-NPs.

**Figure 2:**
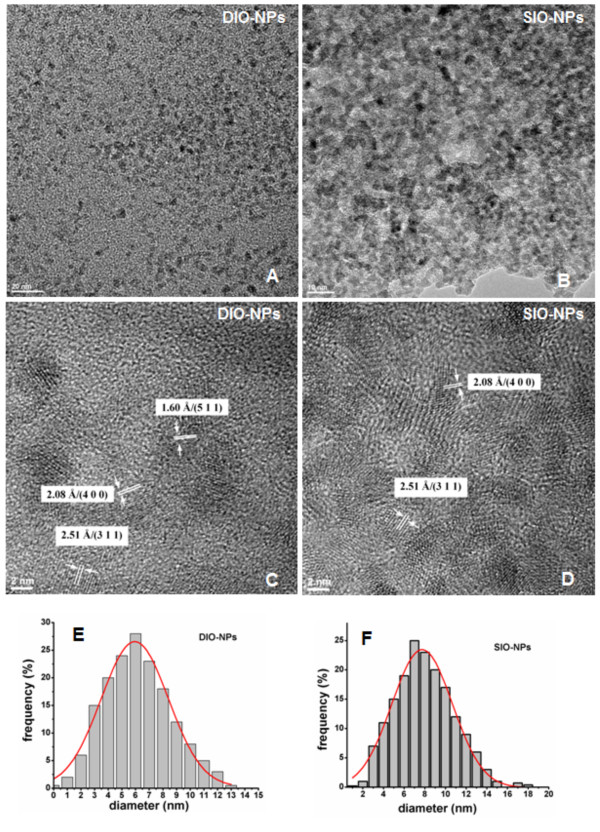
**Large-area TEM and HR-TEM images and size distributions of DIO-NPs and SIO-NPs.** Large-area TEM images of synthesized (**A**) DIO-NPs and (**B**) SIO-NPs. HR-TEM images of (**C**) DIO-NPs and (**D**) SIO-NPs. Size distribution of (**E**) DIO-NPs and (**F**) SIO-NPs.

The diffraction pattern of DIO-NPs and SIO-NPs (Figure 1) shows the peaks that correspond to an fcc maghemite structure (ICSD card no. 01-083-0112) characterized by diffraction planes (220), (311), (400), (422), (511), and (440). No additional diffraction peaks of any impurity were detected, demonstrating the high purity of the synthesized samples. The average particle size was deduced from the full width at half maximum of six lines using Scherrer's relation [28,29]:

(1) $$\begin{array}{l} D\;=\frac{k\lambda }{\beta \;\cos\theta }\\ \beta ={\left(B^{2}|b^{2}\right)}^{\frac{1}{2}} \end{array}$$

where *D* is the averaged length of coherence domains (which is of perfectly ordered crystalline domains) taken in the direction normal to the lattice plane that corresponds to the diffraction line taken into account, *β* is the line broadening due to the small crystallite size, *λ* is the wavelength of X-rays (1.5406 Å), *θ* is the Bragg angle, *B* is the linewidth, and *b* is the instrument line broadening. For linewidths measured as the full width at half maximum peak intensity, *K* is 0.89 [30]. The experimental linewidth was deduced assuming Gaussian profiles for experimental and instrumental broadening in accord with [31]. The line broadening is essentially due to the size effect. The average size, deduced from the full width at half maximum, has a value of 5.8 (±0.5) nm for DIO-NPs and 7.3 (±0.5) nm for SIO-NPs. They are consistent with the mean sizes deduced from high-resolution (HR)-TEM observations (Figure 2). From the *d* value of the peaks, the estimated lattice parameter is 0.835 nm for both samples (DIO-NPs and SIO-NPs), which is consistent with the literature value [32].

The DIO-NPs and SIO-NPs synthesized by the coprecipitation method is shown in Figure 2A,B. The synthesized DIO-NPs and SIO-NPs showed well-shaped spherical nanostructure morphology. Grain size distribution was determined by measuring the mean diameter, *D*, of approximately 500 particles on the micrographs. These monodisperse nanoparticles have an average grain size of 6 nm (DIO-NPs) and 8 nm (SIO-NPs). The grain size distribution was shown in Figure 2E,F. In Figure 2C,D, we show a HR-TEM picture. The clear lattice fringe in the HR-TEM image demonstrates the well crystalline nature of resultant nanoparticles. The interplanar distances (for DIO-NPs) of 2.51, 2.08, and 1.60 Å were attributed to the (311), (400), and (511) planes of maghemite, respectively. In the SIO-NP sample, the interplanar distances of 2.51 and 2.08 Å were consistent with the (311) and (400) planes of maghemite. Therefore, both X-ray diffraction patterns and HR-TEM give feature characteristics of the maghemite structure.

**Figure 3:**
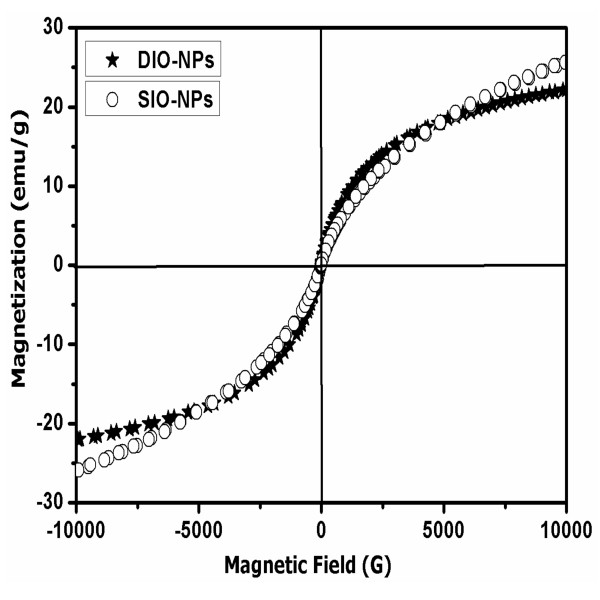
Hysteresis loops measured at 5 K for DIO-NP and SIO-NP samples.

Figure 3 shows magnetization as a function of the applied magnetic field when the magnetization is normalized by the weight of the maghemite nanoparticles. No significant difference was seen among samples both in their superparamagnetic and frozen states. The saturation magnetization (*M*_S_) at 5 K was approximately 22 emu/g for DIO-NPs and 26 emu/g for SIO-NPs. A decreasing size of particles leads to a decrease of *M*_S_ due to increased dispersion in the internal exchange [33]. The surface spin disorder, arising from reduced coordination and broken exchange bonds between surface spins, is expected to give reduced magnetization for samples with smaller diameter.

**Figure 4:**
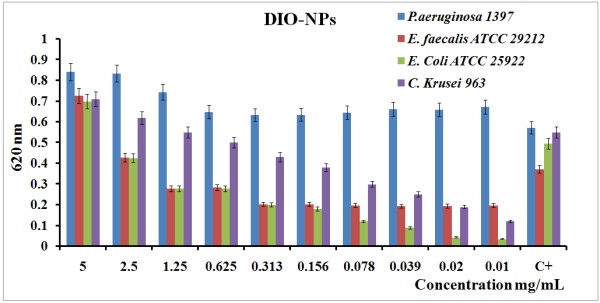
**Antibacterial activity of DIO-NPs using *E. coli, E. faecalis, C. krusei, *and *P. aeruginosa.***

The antibacterial activity of the samples (DIO-NPs and SIO-NPs) was observed using common bacterial pathogens, *E. coli*, *P*. *aeruginosa* (Gram-negative), *E. faecalis* (Gram-positive), and a species of fungus (*C. krusei*). The antibacterial effect of DIO-NPs on the Gram-negative bacteria *E. coli* ATCC 25922 was less visible than that on the Gram-positive bacteria *E. faecalis* ATCC 29212 and *C. krusei* 963 (a species of fungus) for all concentrations. DIO-NPs showed highly significant toxicity to all three bacterial species (Figure 4). The Gram-negative bacteria *P. aeruginosa* 1397 is not inhibited in the presence of DIO-NPs.

**Figure 5:**
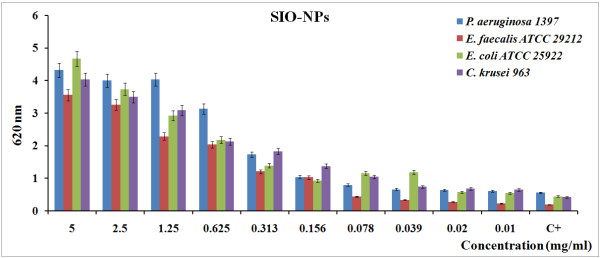
**Antibacterial activity of SIO-NPs using *E. coli, E. faecalis, C. krusei, *and *P. aeruginosa.***

Concerning the effect of DIO-NPs on the microbial growth of the tested strains, we could observe that different concentrations of the tested compound either inhibited or stimulated the growth of *E. faecalis* ATCC 29212, *E. coli* ATCC 25922, and *C. krusei* 963 strains in the suspension. At concentrations lower than 2.5 mg/mL, the DIO-NPs inhibit the growth of the *E. coli* ATCC 25922 strain. The growth of *E. faecalis* ATCC 29212 was inhibited at low concentrations of DIO-NPs (from 0.01 to 1.25 mg/mL). The *C. krusei* 963 strain is inhibited at concentrations between 0.01 and 0.625 mg/mL. The antibacterial activity of DIO-NPS on the Gram-negative bacteria *E. coli* ATCC 2912 was higher than that on the Gram-positive bacteria *E. faecalis* ATCC29212. This is in accord to the previous result using PEGylated ZnO nanoparticles, which exhibited a much stronger antibacterial effect on Gram-negative bacteria [34]. On the other hand, in our study, the SIO-NPs proved to stimulate the growth of microbial cells, as demonstrated by the absorbance measurements at 620 nm of the obtained cultures (Figure 5).

The intensity of the stimulatory effect on the microbial growth proved to be proportional with the concentration of SIO-NPs, as proved by the linear trend lines. In exchange, all tested concentrations of DIO-NPs and SIO-NPs slightly stimulated the growth of *P. aeruginosa* 1397*.* In general, the stimulatory effect on the microbial growth was higher for sucrose than for dextran. These results may be due to the fact that natural sucrose has a vital role as a transport carbohydrate and sometimes also as a storage carbohydrate. Wu and Birch [35] showed that some microbes convert sucrose with remarkable yields into the structural isomers isomaltulose and trehalulose, possibly to sequester the sugar in a form that confers an advantage against competing species. On the other hand, the use of sucrose isomers is currently limited by the expense of microbial or enzymatic conversion from more abundant plant-derived sucrose [36]. The last studies demonstrated that sucrose content of pulp from the roots decreased when the number of bacteria increased under anaerobic storage. A difference in the antimicrobial activity of DIO-NPs and SIO-NPs may come from active oxygen species generated by the powder in solution. Indeed, every bacterium responds unevenly to oxidative stress due to differences in the permeability of cell membranes [37]. Some microbial strains succumb to damage to cell walls by O^2−^ and others, while others show greater sensitivity to H_2_O_2_, as is the case for *E. coli*[38]. Shi et al. [39] showed that the increase of antibacterial activity is directly related to the increase of active oxygen generated on the surface of particles of oxide nanoparticle, reducing the size of the particle. Moreover, the nanoparticle of oxides in solution enhances the possibility of interaction between the particle and the bacterial cell due to its surface charge and surface energy [38,40].

Finally, it is important to emphasize that the antimicrobial activity of the DIO-NPs was more potent than that of the SIO-NPs. NP bacterial interactions are influenced by interfacial forces, especially electrostatic, that can control the interaction between NPs and the bacterial surface [41]. The results showed that antibacterial DIO-NPs were performed by attaching dextran to iron oxide using a coprecipitation method. Importantly, the antibacterial activities are due to the surfactant from the surface of the iron oxide nanoparticles. The hydroxyl groups present in dextran offer many sites for derivatization, and these functionalized glycoconjugates represent a largely unexplored class of biocompatible and environmentally safe compounds. Therefore, it could be concluded that dextran-coated iron oxide nanoparticles could be released through aqueous carbohydrate solutions owing to the stable dispersion at molecular level and the slow diffusion from the stabilizing medium [42]. The mechanism of iron oxide NP antibacterial activity and the properties related to toxicity are still not clearly understood.

## Conclusions

The polysaccharide-coated maghemite prepared under simple adapted chemical method has a particle size considerably smaller than that reported in the literature. The synthesized DIO-NPs and SIO-NPs containing only maghemite in crystalline phase showed spherical well-shaped nanostructure morphology. The *M*_S_ at 5 K of DIO-NPs and SIO-NPs was approximately 22 emu/g for DIO-NPs and 26 emu/g for SIO-NPs.

In the current paper, we also showed that yeast *P. aeruginosa* 1397 was resistant to DIO-NPs and SIO-NPs. Antimicrobial activity of the DIO-NPs and SIO-NPs is influenced by the polysaccharide type and the size of the particles. The bacterial sensitivity to each type of NP also varied with the bacterial strain. The antibacterial activity of PMC-NPs based on dextran was significantly higher than that of PMC-NPs based on sucrose for *E. faecalis* ATCC 29212, *C. krusei* 963, and *E. coli* ATCC 25922.

Further work is needed to study the effect of antibacterial activity of iron oxide nanoparticles with different sizes and coated with different biopolymers. The present investigation would therefore facilitate future antibacterial studies on iron oxide nanoparticles and prove to be an important step in the exploration of nanoparticles for other microbial processes in commercial utility.

## Competing interests

The authors declare that they have no competing interests.

## Authors’ contributions

DP conceived the study. SLI and AMP performed the synthesis of the samples. Characterization of materials was carried out by DP, SLI, AMP, MM, and SS. SLI performed the antibacterial investigations. MM and SS performed the magnetic investigations. DP directed the study and wrote the draft paper. All authors contributed to the interpretation of results and discussion and have corrected, read, and approved the final manuscript.
